# Gastric-type mucinous endocervical adenocarcinomas: A case report and literature review

**DOI:** 10.3389/fcimb.2022.917009

**Published:** 2022-10-13

**Authors:** Junling Lu, Jing Na, Ya Li, Xinyou Wang, Jun Wang, Shichao Han

**Affiliations:** Department of Gynecology and Obstetrics, Second Affiliated Hospital of Dalian Medical University, Dalian, China

**Keywords:** human papillomavirus, gastric type, adenocarcinoma, mucinous, diagnosis, therapy

## Abstract

Gastric-type mucinous endocervical adenocarcinomas (GAS) are new variant types of cervical adenocarcinomas according to the 2014 World Health Organization (WHO) classification. GAS is a unique disease that can be differentiated from typical adenocarcinomas—it is less common and more aggressive and likely to have deep invasion and horizontal diffusion, invasion of the uterus and vagina, early distant metastases, and a lower 5-year survival rate compared to the usual-type cervical cancer. At present, initial treatment and postoperative adjuvant therapy are not conclusive, but early detection and early treatment are a consensus that can improve prognosis. Most of its occurrence has nothing to do with human papillomavirus (HPV) infection. Whether it is only negative for the subtypes that can be detected at present and whether it may be an unknown subtype of infection need to be further explored in the future. The clinical symptoms commonly include aqueous secretion, lower abdominal pain, and elevated serum carbohydrate antigen-19-9 (CA19-9) levels, which may be helpful for diagnosis. MRI and PET-CT can help to describe the characteristics of lesions and judge the state of the systemic metastasis. We believe that early detection and surgical treatment will give patients more benefits. Looking for potential gene and molecular changes and establishing biomarkers to identify molecular targets will be the key to early identification and target therapy.

## Introduction

Cervical cancers are the fourth most frequent cancers among women in the world (Ferlay et al., 2015), which are the most common reasons for cancer deaths in women globally. In 2020, the World Health Organization (WHO) proposed a global strategy, which is expediting the elimination of cervical cancer as a community health issue (WHO, 2020). As we all know, infection with high-risk human papillomavirus (HPV) has been implicated as the primary cause of cervical carcinogenesis (Schiffman et al., 2007). However, we found that few patients with cervical cancer are HPV negative. What is terrible is that these patients are easy to be forgotten and missed. We will report a case of specific tumor stage IIIc1p gastric-type mucinous endocervical adenocarcinoma (GAS) that is HPV negative. Meanwhile, by searching PubMed, ClinicalKey, and other databases, we try to retrospectively analyze the related research of GAS in the recent 10 years, which is expected to summarize the experience of diagnosis and treatment of GAS.

## Case report

A 42-year-old woman was transferred to our hospital in January 2022. Approximately 5 months before hospitalization, the patient began to have no obvious inducement to discharge a large amount of transparent thin mucus from the vagina without peculiar smell. The symptoms were relieved after vaginal medication. Two weeks before hospitalization, she developed a dull pain in her lower abdomen also without an apparent cause. At that time, she had no fever, dizziness, fatigue, and other discomfort. She accepted the ultrasound examination at the local community clinic that showed that the local echo of the cervix was reduced and the blood flow at the external orifice of the cervix was abundant. Subsequently, she accepted ThinPrep cytologic test (TCT) and HPV genotype test in the local hospital. The results of the TCT showed atypical glandular cells (AGCs), and the HPV DNA test was negative. Then, she underwent colposcopy, cervical multipoint biopsy, and endocervical curettage. The pathology showed mucinous adenocarcinoma and gastric adenocarcinoma. Finally, she came to our hospital in order to seek further diagnosis and treatment. She is in good health and has no chronic and infectious diseases in the past. She had never been screened for cervical cancer or vaccinated against cervical cancer before. She has been pregnant eight times, including two cesarean sections and six abortions. There is no history of cancer in her family. Her mother and sister are all suffering from hypertension, and her father is suffering from diabetes.

**Figure 1:**
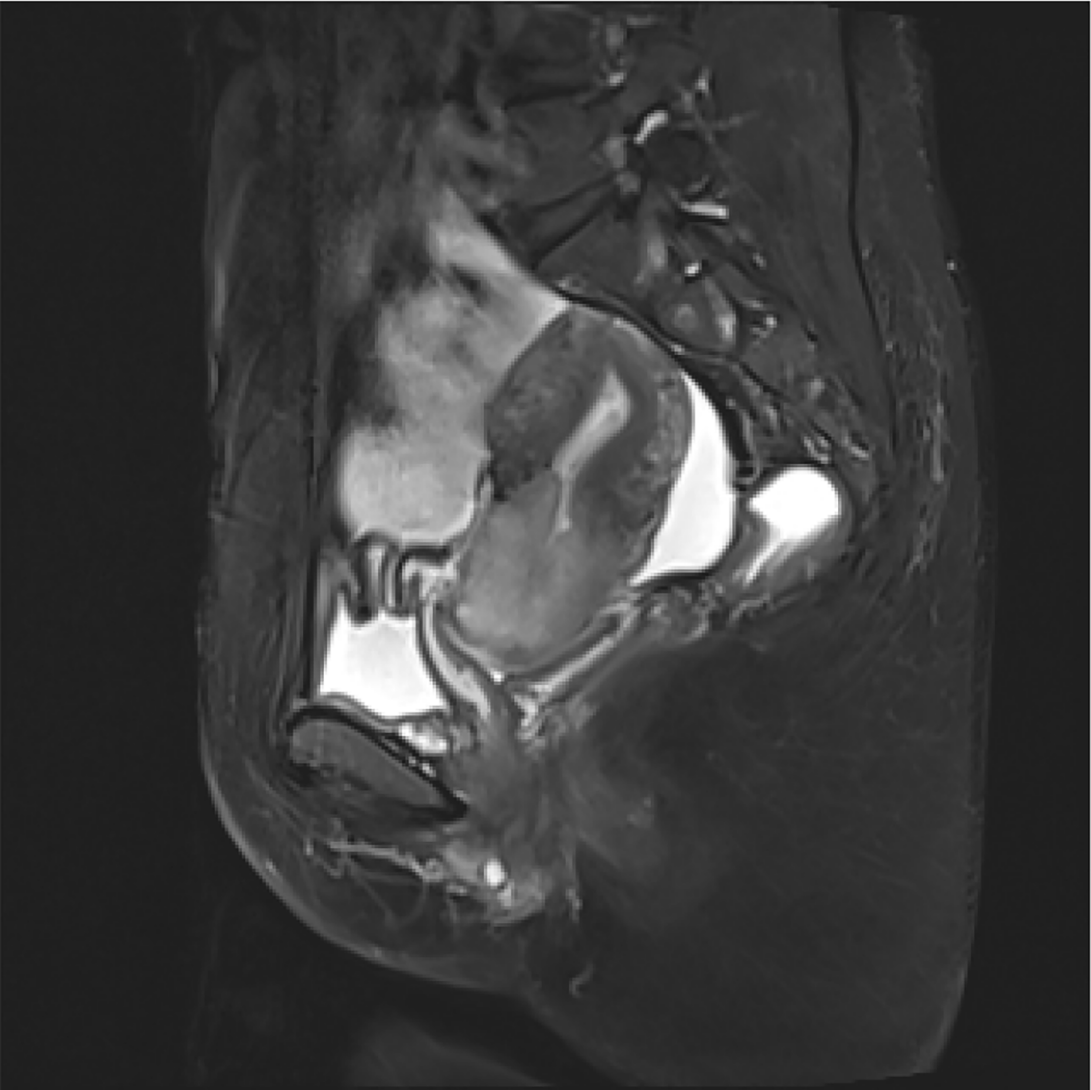
Pelvic magnetic resonance imaging (MRI) showed a cervical tumor 40 mm * 36 mm * 48 mm in size, without invasion of the uterus and vagina, and no pelvic lymph node metastasis.

**Figure 2:**
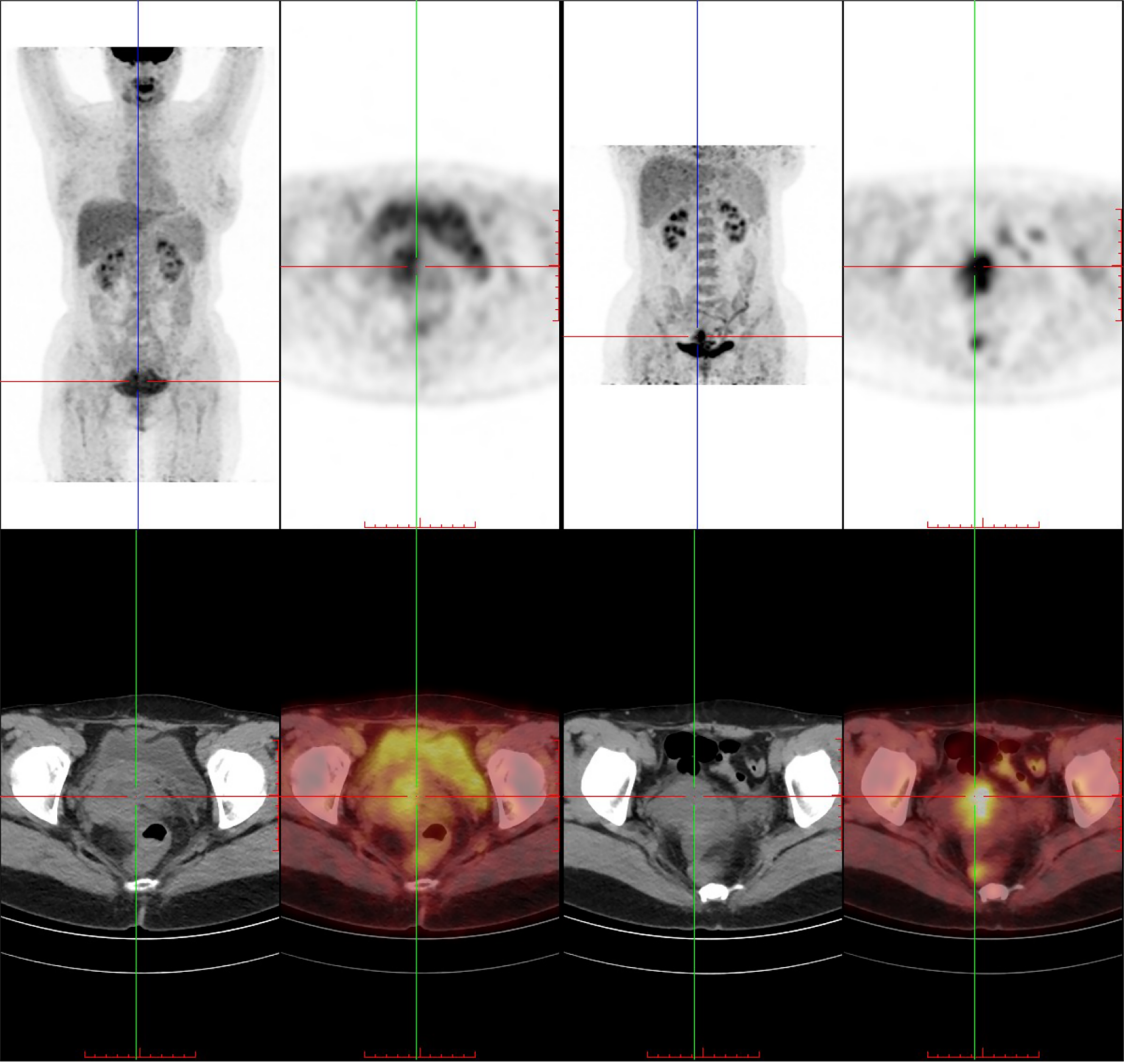
The cervix is widened with mass radioactive concentration foci, with a range of about 38 mm * 24 mm, SUVmax 4.0, delay 5.2. Radioactive concentration can be seen in the lower part of the uterine body, SUVmax 3.9, delay 5.6. Uterine effusion.

**Figure 3:**
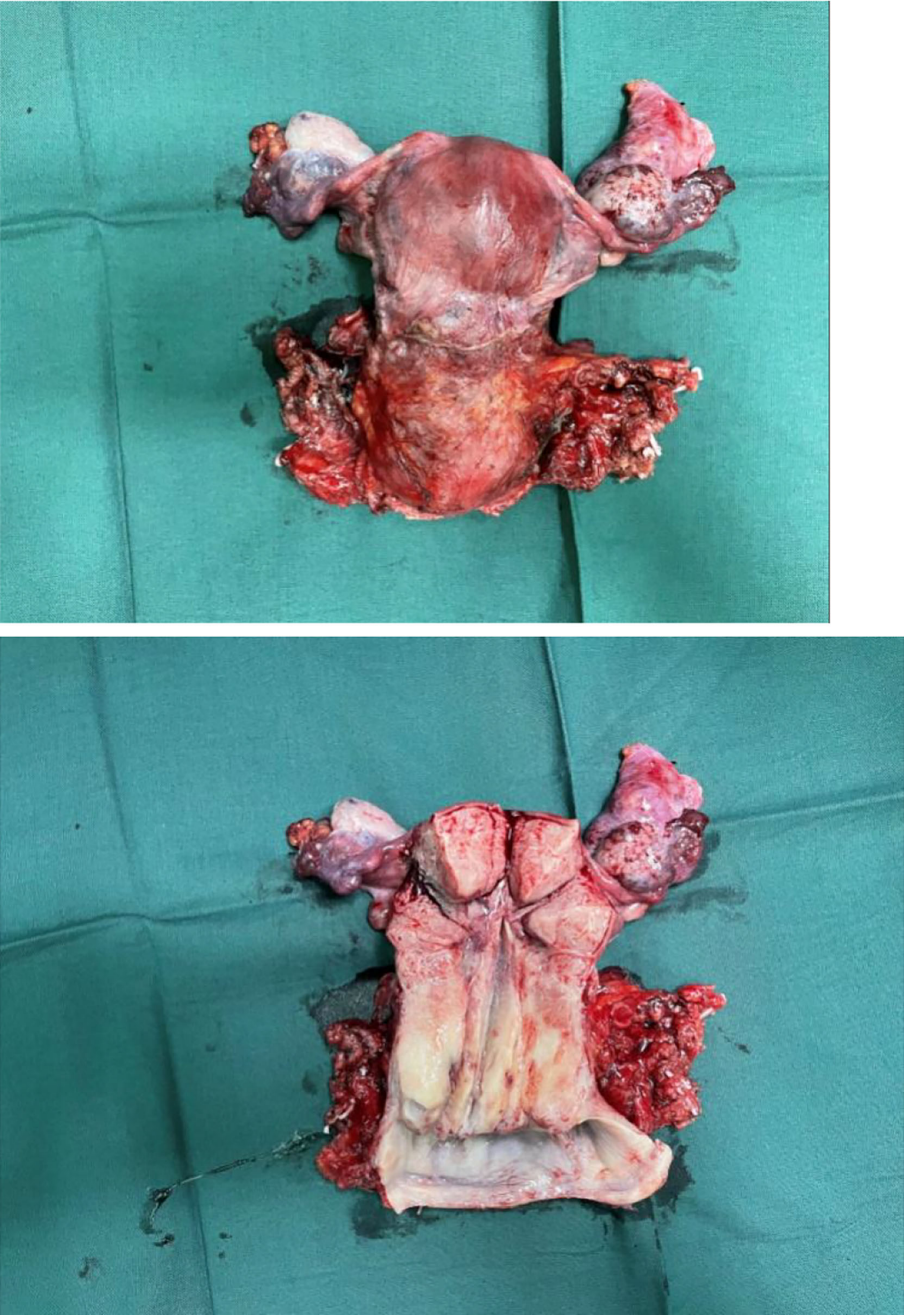
Isolated uterus after the operation.

**Figure 4:**
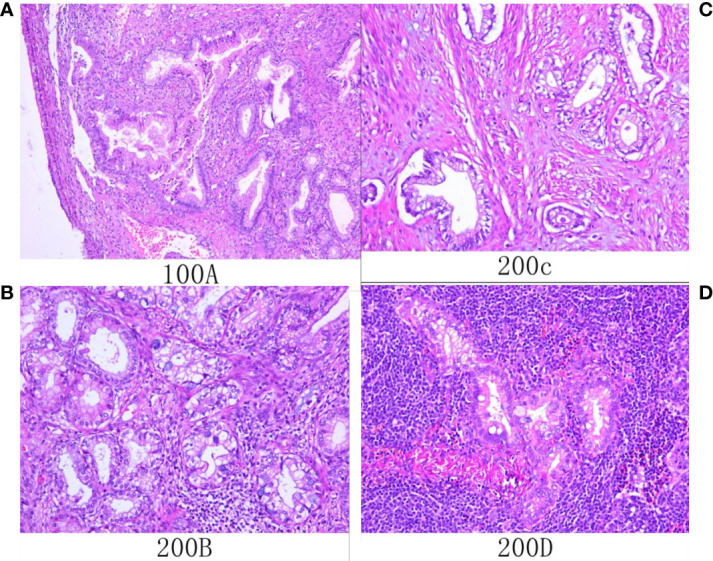
Histological (**A–D**; hematoxylin and eosin staining) results. **(A)** Microscopic examination showed that atypical glands are irregular and expand with gastric differentiation. **(B)** The tumor showed gastric differentiation, including an atypical nucleus and abundant eosinophilic cytoplasm that was transparent or pale. **(C)** The tumor invaded the myometrium. **(D)** Tumor cells metastasized to local lymph nodes.

By bimanual rectovaginal examination, we found that the cervical surface was smooth but barrel thickened, which is easy to bleed when touched. At the same time, we did not find parametrial invasion. MRI of the pelvis showed that the cervical mass was about 40 mm * 36 mm * 48 mm, invading the lower part of the uterine body and pelvic vessels, but without parametrial, vaginal extension and enlarged pelvic lymph nodes (**Figure 1**). PET-CT showed that there were a large number of radioactive foci in the cervix and lower uterine segment, which were suspected to be malignant tumors, but there was no obvious radioactive foci, suggesting lymph node metastasis in the pelvic cavity and distant metastasis (**Figure 2**). Carbohydrate antigen 125 (CA125), carcinoembryonic antigen (CEA), alpha fetoprotein (AFP), and human epididymis protein 4 (HE4) were all within normal range, but carbohydrate antigen-19-9 (CA19-9) and the antigen of squamous cell carcinoma (SCC) rose; the corresponding results were 1,303.61 U/ml and 2.89 ng/ml, especially CA19-9 increased significantly. No obvious abnormality was found in other examinations including colonoscopy and gastroscopy. Finally, according to the clinical staging standard of the International Federation of Obstetrics and Gynecology (FIGO, 2018), we set the preoperative staging of the patient as IB3. Then, we performed cystoscopy, bilateral ureteral D-J tube placement, extensive hysterectomy (type C), bilateral salpingo-oophorectomy, and bilateral pelvic lymphadenectomy (level 2). After surgery, the isolated uterus was dissected. The morphology of the uterus was regular. No space-occupying lesions were found in the uterine cavity. The endometrium was thin. Tumors could be seen between the circumferential walls of the cervix. The size was about 4 cm * 4 cm * 3.5 cm and did not invade the vagina. According to the naked eye examination of the sample, the deep cervical interstitial infiltration was full-thickness. The width of the parauterine tissues on both sides was measuredto be about 3 cm, the length of the vaginal resection was about 3 cm, and there was no significant increase in pelvic lymph nodes on both sides (**Figure 3**). The pathology revealed that the cervical adenocarcinoma (non-HPV-related, gastric-type), with an infiltration depth of 1.7 cm, infiltrates the whole layer of the cervical muscle wall. Multiple intravascular tumor thrombi and nerve invasion can be seen, involving the endometrium and the muscle layer, without involving the vaginal fornix. No cancer tissue was found in the broken end of the vagina and parauterine soft tissue. Two of the 20 pelvic lymph nodes showed metastasis and invasion (**Figure 4**). Immunohistochemical stains showed that tumor cells were MLH1 (+), MSH (+),PMS2 (+), HER2 (1+), ER (<1% weak +), BRCA1 (+), p53 (85% medium intensity +), ERCC1 (+), PRMI (–), TopoII grade. During molecular detection, no mutation of BRAF gene V600E was detected. On the first day after the operation, CA19-9 decreased rapidly (662.16 µ/ml) and fell to the normal range (CA19-9: 12.72 µ/ml) after 1 month. Because she has some prognostic factors, such as tumor body 4 cm and involves the whole layer of the cervix, multiple intravascular tumor thrombi and nerve invasion, partial uterine body involvement and pelvic lymph node metastasis, she needs to receive a combination of chemotherapy and radiotherapy. We plan that she will receive three cycles of chemotherapy first, then pelvic radiotherapy, and finally three cycles of chemotherapy.

## Discussion

Endocervical adenocarcinoma (ECA) is a group of tumors with higher heterogeneity, and about 15% is not associated with HPV infection (Stolnicu et al., 2018). In 2014, WHO classified cervical adenocarcinoma according to morphological and cellular characteristics and proposed a new disease category of GAS for the first time (Kurman et al., 2014). In 2018, the International Endocervical Adenocarcinoma Criteria and Classification (IECC) system attempted to categorize ECA by etiology (HPV status) and morphology with molecular and outcome evidence (Stolnicu et al., 2018). Obviously, the latter is more comprehensive and clinically relevant. It is reported that the incidence rate of GAS is 20%–25% of all cervical adenocarcinomas (Mikami and McCluggage, 2013).

HPV infection is directly related to cancer. High-risk HPV is highly associated with genital cancers including cervical cancer and head and neck squamous cell carcinoma (HNSCC) (Antonsson et al., 2015; Schiffman et al., 2016). Data showed that HPV was detected in 99.7% of cervical cancer patients, in whom HPV16 alone accounted for about 55% (de Sanjosé et al., 2007). The incidence rate of HNSCC caused by HPV infection has been increasing in recent years (Morgan et al., 2017). Several studies on cervical adenocarcinoma have shown that the prevalence of HPV infection is lower. About 5%–25% of cervical adenocarcinoma is independent of HPV, which depends on many factors such as geographical region, histological subtype, and HPV detection method (Pirog et al., 2014; Molijn et al., 2016). Several studies have indicated that GAS is mainly not related to HPV infection (Houghton et al., 2010; Park et al., 2011). In addition, whether it is only negative for the subtypes that can be detected at present and whether it may be an unknown subtype of infection need to be further explored in the future. In the era of HPV vaccination, the prevention and screening of cervical cancer have played a vital role. For HPV-negative cervical gastric adenocarcinoma, we still need to further explore its pathogenesis and methods of prevention and screening.

Early detection and timely treatment of cervical precancerous lesions are very important for cancer prevention and early detection. The high-risk HPV test of patients with GAS is usually negative, while the positive rate of routine cervical exfoliative cytology is low, which poses a great challenge for us to find its precursor lesions. The precursor lesions of GAS include lobar endocervical glandular hyperplasia (LEGH), atypical LEGH, and endocervical adenocarcinoma *in situ* (AIS). The latter two have common genetic characteristics, such as 1p deletion and 3q acquisition. In addition, GAS is also closely related to Peutz-Jeghers syndrome (PJS) (Kawauchi et al., 2008; Beggs et al., 2010). About 10% of GAS patients have PJS, which may be related to STK11 gene mutation (Peng et al., 2015). These may bring some help to our early discovery of GAS.

As early as 1988, a case–control study of 39 cases and 409 controls in the greater Milan region showed that the relative risk of cervical adenocarcinoma increased with the increase of the number of deliveries and abortions, the age of birth, and the age of first sexual intercourse (Parazzini et al., 1988). The current analyses were based on data from the multicenter TeQaZ study, which shows that currently living with a partner, having at least four children, having had more than one sexual partner, ever smoking, and obesity were significantly associated with cervical cancer (Tanaka et al., 2021). In our report, the patient has been pregnant eight times, including two cesarean sections and six abortions. We think that multiple pregnancies may be associated with cervical cancer, but whether it is a high-risk factor for the occurrence of GAS still needs further research to confirm.

Nakamura et al. (2018) reviewed 322 cases of cervical cancer and found that it is hard to diagnose GAS by preoperative biopsy, but some specific clinical manifestations, such as aqueous secretion and lower abdominal pain, high serum CA19-9 level, and immunohistochemistry (HIK1083 and MUC6 staining), often help the diagnosis. Ihara et al. (1988) showed that CA125 and CA19-9 are useful tumor markers for the treatment of cervical adenocarcinoma and endometrial cancer, especially in advanced or recurrent cases. They found that CA125 or CA19-9 immunohistochemistry was localized in cancer tissue, and in most recurrent cases, serum CA125 and CA19-9 increased significantly in the early stage (Ihara et al., 1988). Maruyama et al. (1988) used histochemical methods to detect the immunohistochemical localization of epithelial mucin and carbohydrate antigen (CA125, CA19-9) in normal cervical glands and cervical adenocarcinoma. They found that CA19-9, CA125, and CEA were localized in cervical adenocarcinoma and intestinal adenocarcinoma groups (Maruyama et al., 1988). The patient in our case also had more typical symptoms of vaginal fluid and lower abdominal pain. The character of vaginal discharge in patients with gastric adenocarcinoma is the same as that of gastric secretion, and the mechanism remains to be further explored. In our case, CA19-9 increased significantly before the operation, decreased to one-half on the first day after the operation, and returned to normal level 1 month after the operation. Combined with previous studies, we believe that CA19-9 may be used as a reference index to monitor the efficacy and recurrence of gastric cervical adenocarcinoma. At the same time, MRI is a good and accessible tool for diagnosis, which can describe the characteristics of lesions and evaluate local regional expansion. PET-CT can help judge the state of the whole body and whether there is distant metastasis. In our case, the tumor cells were with much transparent and light eosinophilic cytoplasm, clear cell boundaries, low nuclear plasma ratio, and irregularly distributed nuclei at the base of the gland. It is consistent with the pathomorphological characteristics of cervical gastric adenocarcinoma.

Several recent studies have revealed diversities in the pathological mechanism and molecular features between GAS and usual-type endocervical adenocarcinoma (UEA). They found that GAS showed obviously deeper infiltration depth, greater horizontal diffusion, later stage, and more common distant metastasis. In other words, it was more likely to invade the parauterine and vagina. At the same time, P53 protein was always immunostained. Moreover, the disease-free survival time of GAS was obviously shorter than that of UEA (Nishio et al., 2019; Jung et al., 2020). In our case, the patient was diagnosed as IB3 before the operation, but postoperative pathology confirmed pelvic lymph node metastasis, which is basically consistent with their findings. Garg et al. (2019) elucidate the molecular characteristics of gastric cervical adenocarcinoma by using next-generation sequencing (NGS) to evaluate 161 unique cancer-driver genes. Their study showed the genetic heterogeneity of gastric cervical adenocarcinoma and found that there may be some potentially operable molecular changes. These molecular characteristics are very important for better discerning this rare tumor and better treatment (Garg et al., 2019). Lu et al. (2021) also used NGS technology to identify the unique genomic changes of GAS, mainly involving cell cycle and PI3K/AKT (Phosphatidylinositde-3-Kinase/Protein Kinase B) signaling pathways. If we can further identify the molecular characterization of GAS and find out the potential operable molecular changes, it will bring new hope for tumor-targeted therapy. By finding genetic changes of the disease, we can understand the pathogenesis better, and it is possible to get potential targets and individualized treatment strategies in the future.

As GAS is relatively rare, there is still lack of prospective clinical research. The treatment of GAS is controversial, and there is no unified standard. The Chinese Expert Consensus on the Clinical Diagnosis and Treatment of Cervical and Gastric Adenocarcinoma (2021 Edition) pointed out that in the absence of standard treatment methods, the treatment of GAS can refer to the 2022 NCCN Clinical Practice Guidelines for Cervical Cancer (First Edition) for the treatment of small cell carcinoma (Gynecological oncology professional committee (Study Group) of gynecologist and obstetrician branch of Chinese Medical Association, 2021). Therefore, the selection of our case treatment plan is based on NCCN guidelines. This guideline points out that for patients with tumors ≤4 cm, radical hysterectomy + pelvic lymphadenectomy ± para-aortic lymph node sampling is the first choice for surgery. Chemotherapy or concurrent radiotherapy and chemotherapy can be selected after the operation. However, you can also choose concurrent radiotherapy and chemotherapy + brachytherapy and then consider combining other systemic treatments (NCCN, 2022). Whether the scope of surgery is similar to that of ovarian cancer staging is still controversial. We believe that whether bilateral appendectomy, omentum resection, and appendectomy can prolong the survival time and improve the quality of life of patients still needs further study. It is reported that gastric cervical adenocarcinoma has chemoresistance. Therefore, its prognosis is poorer than the other types of cervical adenocarcinomas (Kawakami et al., 2010). Li et al. (2010) emphasized the importance of surgical treatment, followed for advanced stages by radiochemotherapy. Therefore, we believe that early detection and surgical treatment will give patients more benefits.

The recurrence rate of GAS is higher, approximately 40%, and the 5-year survival rate is only about 30% (Kojima et al., 2007; Nishio et al., 2019). The prognosis is related to tumor stage, parauterine invasion, resection margin, metastasis, and treatment. A study found that patients with programmed death-ligand 1 (PD-L1)-positive GAS had a worse prognosis (including disease progression-free survival and overall survival) than those with PD-L1-negative GAS. It is suggested that PD-L1 can be used as a marker of poor prognosis of GAS (Chen et al., 2021). We will closely follow up this patient and continue to track her prognosis. In the future, we need to do more work to find indicators to monitor the prognosis of GAS.

## Conclusions

GAS is usually HPV negative, which is difficult to find in cervical precancer screening. Although the current literature reports that GAS is not related to high-risk HPV (Kojima et al., 2007; Kurman et al., 2014; Mikami et al., 2020), whether it is only negative for the currently detectable subtypes and whether it may be an unknown infection subtype are worth further exploration in the future. Combined with previous studies, we think that CA19-9 may be used as a reference index to monitor the efficacy and recurrence of gastric cervical adenocarcinoma. It is not sensitive to chemotherapy drugs, and the prognosis is worse than those of SCC and cervical adenocarcinoma. Early radical surgery is very important for patients. Looking for potential gene and molecular changes and establishing biomarkers to identify molecular targets will be the key to early detection and targeted therapy.

## Data availability statement

The original contributions presented in the study are included in the article/supplementary material. Further inquiries can be directed to the corresponding authors.

## Ethics statement

Written informed consent was obtained from the individual(s) for the publication of any potentially identifiable images or data included in this article.

## Author contributions

JL and SH dealt with the case and drafted the manuscript. JN and YL assisted collected case data and literature and carried out all the documentary and article work out. JW has made great efforts and has given many constructive suggestions for this paper during the revision and production period. All authors contributed to the article and approved the submitted version.

## Conflict of interest

The authors declare that the research was conducted in the absence of any commercial or financial relationships that could be construed as a potential conflict of interest.

## Publisher’s note

All claims expressed in this article are solely those of the authors and do not necessarily represent those of their affiliated organizations, or those of the publisher, the editors and the reviewers. Any product that may be evaluated in this article, or claim that may be made by its manufacturer, is not guaranteed or endorsed by the publisher.
